# Endovascular Mechanical Thrombectomy and On-Site Chemical Thrombolysis for Severe Cerebral Venous Sinus Thrombosis

**DOI:** 10.1038/s41598-020-61884-5

**Published:** 2020-03-18

**Authors:** Chih-Hsiang Liao, Nien-Chen Liao, Wen-Hsien Chen, Hung-Chieh Chen, Chiung-Chyi Shen, Shun-Fa Yang, Yuang-Seng Tsuei

**Affiliations:** 1 Institute of Medicine, Chung Shan Medical University, Taichung, Taiwan; 2 Department of Neurosurgery, Neurological Institute, Taichung Veterans General Hospital, Taichung, Taiwan; 3 Department of Critical Care Medicine, Taichung Veterans General Hospital, Taichung, Taiwan; 4 Department of Neurology, Neurological Institute, Taichung Veterans General Hospital, Taichung, Taiwan; 5 Division of Neuroradiology, Department of Radiology, Taichung Veterans General Hospital, Taichung, Taiwan; 6 School of Medicine, National Yang-Ming University, Taipei, Taiwan; 7 Department of Neurosurgery, Tri-service General Hospital, National Defense Medical Center, Taipei, Taiwan; 8 Department of Medical Research, Chung Shan Medical University Hospital, Taichung, Taiwan; 9 Department of Physical Therapy, Hung Kuang University, Taichung, Taiwan; 10 Basic Medical Education Center, Central Taiwan University of Science and Technology, Taichung, Taiwan

**Keywords:** Brain, Stroke, Outcomes research, Stroke

## Abstract

Cerebral venous sinus thrombosis (CVST) is a rare cause of cerebral infarction. Once patients survive the acute phase, long-term prognosis is generally satisfactory. CVST patients who harbored risk factors known for poor prognosis (e.g., deterioration of consciousness/neurological functions and seizures) were oftentimes unresponsive to systemic heparin treatment. The advantage of combined endovascular mechanical thrombectomy (EMT) and on-site chemical thrombolysis (OCT) plus systemic heparin for CVST over the heparin treatment alone has not been proved. A retrospective study was conducted to analyze consecutive patients with CVST from 2005 to 2015. Patients having clinical improvement or stable disease after heparin treatment were in I/S group; patients having continuous deterioration of consciousness/neurological functions and refractory seizures (despite the use of multiple anti-epileptic drugs) after heparin treatment were in D group. EMT and OCT were indicated for patients in D group. Imaging studies and medical records were reviewed for statistical analysis. Safety issues included new-onset/progression of symptomatic intracerebral hemorrhages (ICH) or procedure-related complications. Total thirty patients were included (I/S group = 16; D group = 14). In D group, the mean time frame from the start of heparin treatment to the endovascular treatment was 3.2 days. Compared with I/S group, all patients in D group had complete stenosis of the sinuses, with higher initial mRS, lower initial GCS, and more seizures (p = 0.006, 0.007, and 0.031, respectively), but no significant differences in the mRS at discharge (p = 0.504). Shorter length of thrombosis and lower initial mRS were associated with better outcomes (p = 0.009 and 0.003, respectively). Thrombosis involving the superior sagittal sinus (SSS) was associated with bad outcomes (p = 0.026). There were two patients (6.7%) with worsening symptomatic ICH, one in each group, managed surgically. The overall mortality of the study was 6.7% (2/30). Combined EMT and OCT after heparin treatment for severe CVST were reasonably safe, which might be considered as a salvage treatment in severe CVST patients who are unresponsive to heparin with heavy clot burden involving SSS in the acute phase. However, further studies are needed to confirm its efficacy and validity.

## Introduction

Cerebral venous sinus thrombosis (CVST) is a rare disease. The diagnosis could easily be missed if the initial presentation is non-specific headaches. Heparin (unfractionated heparin or low-molecular-weight heparin) is the first-line treatment. Concomitant intracerebral hemorrhage related to CVST is not a contraindication to heparin therapy^1–3^. Once the patient survived the acute phase, long-term prognosis was generally satisfactory^4^. According to the ISCVT study, despite best medications, 13.6% of patients still had bad outcomes (mRS = 2–5), and the mortality rate was 8.3% after an average follow-up of 16 months^4^. Endovascular treatment might provide theoretical benefits to achieve direct recanalization. However, no statistical data showed additional benefit of the endovascular treatment over heparin in the acute phase because of the rarity of the disease^2,3^. In this study, the authors compared the heparin responders with the heparin non-responders, who received additional endovascular treatment, and analyzed their clinical characteristics and outcomes in the acute phase.

## Clinical Materials and Methods

### Study design

The authors conducted a retrospective study of consecutive CVST patients in a single center from January 2005 to December 2015. The following data of the patients with confirmed diagnosis of CVST were extracted from the medical records: age, gender, onset time, admission modified Rankin Scale (mRS)/Glasgow Coma Scale (GCS), precipitating factors, and mRS/GCS at discharge and 3-months follow-up, etc. Patients with sinus occlusion/thrombosis due to direct traumatic injury, direct tumor compression, or septic emboli were excluded. Demographic, clinical characteristics and imaging studies were reviewed and analyzed. This study was approved by the Institutional Review Board I&II of Taichung Veterans General Hospital (IRB TCVGH No.: CE17084A), and the board waived the need for patient informed consent due to the retrospective nature of this study.

### Treatment strategy & grouping

**Figure 1:**
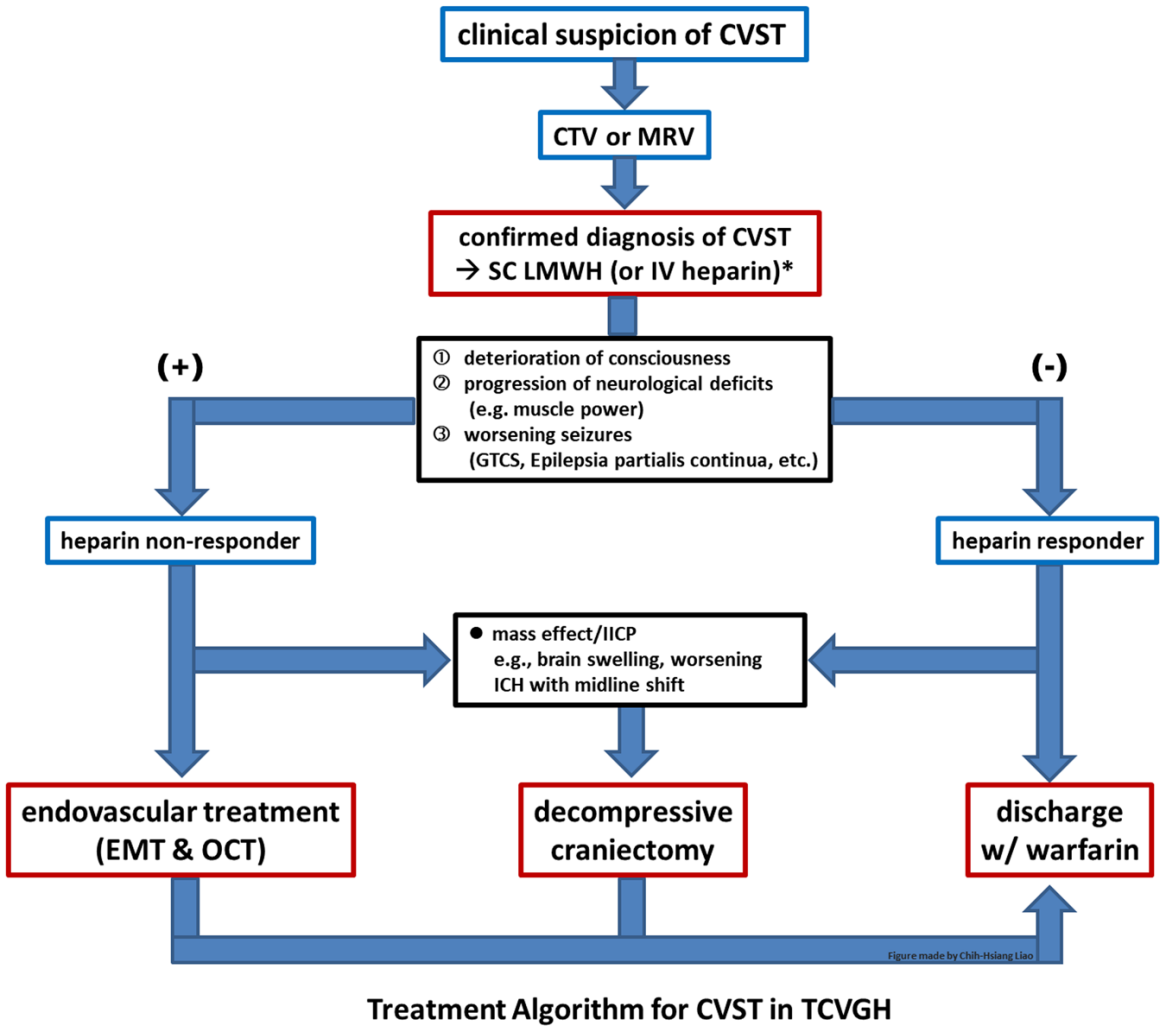
Treatment algorithm of CVST patients in the authors’ institute. Heparin non-responders who have high risks of poor outcome/mortality receive additional endovascular treatment. Decompressive craniectomy was performed in CVST patients with life-threatening conditions (e.g. brain swelling with midline shift, the progression of ICH). *Adjusted-dose UFH or weight-based LMWH was used.

In our institute, after the diagnosis of CVST was confirmed, all of the patients received heparin treatment (adjusted-dose UFH or weight-based LMWH), regardless of the presence of intracerebral hemorrhage (ICH)^1–3^. The treatment protocol of UFH was: loading dose of 80 units/kg, followed by 18 units/kg/hr with continuous intravenous infusion and dosage adjustment to achieve a target activated partial-thromboplastin time of 60–85 seconds), and that of LMWH was: enoxaparin 1 mg/kg subcutaneously twice a day. In patients who received UFH, aPTT levels were monitored every six hours, and patients who received LMWH did not require aPTT monitoring. Patients having clinical improvement or stable disease after heparin treatment were defined as clinical improving/stable patients (I/S group). Patients having (1) continuous deterioration of consciousness, (2) progression of neurological deficits (e.g. muscle power), or (3) worsening seizures (generalized tonic-clonic seizures, epilepsia partialis continua, etc., despite the use of multiple anti-epileptic drugs) after heparin treatment, which were poor prognostic factors in CVST patients according to the literature^5–9^, were defined as clinical deteriorating patients (D group). The treatment algorithm for CVST in the authors’ institute was summarized in Fig. 1. The endovascular treatment (mechanical thrombectomy and on-site chemical thrombolysis) was indicated for patients in D group. Decompressive craniectomy was performed in CVST patients with life-threatening conditions (e.g. brain swelling with midline shift, the progression of ICH) after repeated imaging studies.

### Diagnosis, classification, and imaging evaluation

**Figure 2:**
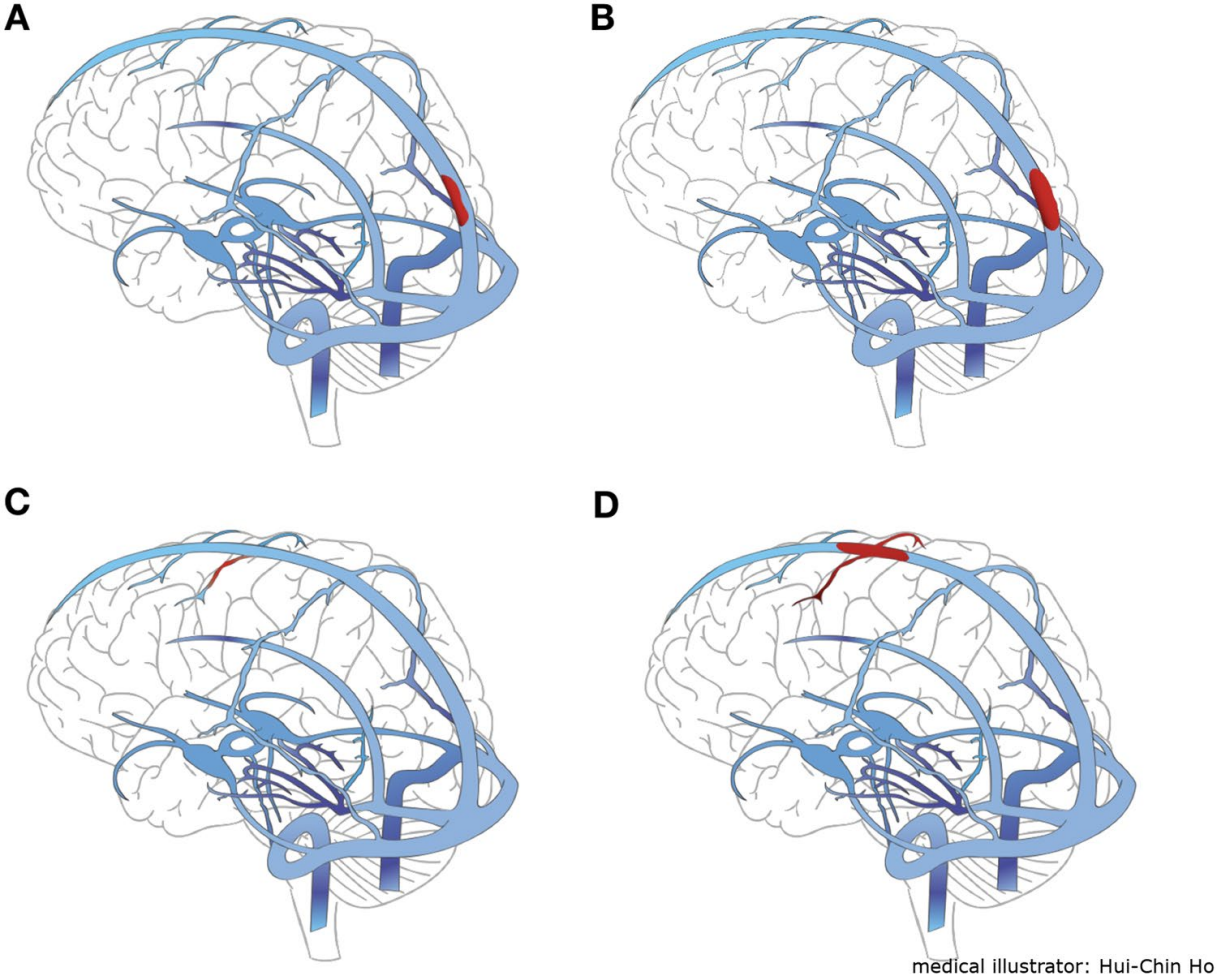
A classification for CVST configurations. The sites of thrombosis and blood clots were in red; unaffected sinuses and cortical veins were in blue. (**A**) Type A, partial thrombosis of the sinus. (**B**) Type B, complete thrombosis of the sinus without cortical vein involvement. (**C**) Type C, cortical vein thrombosis only. (**D**) Type D, complete thrombosis of the sinus with cortical vein involvement.

The diagnosis of CVST was suspected from the clinical presentation and confirmed with computed tomography venography (CTV) and magnetic resonance venography (MRV) that demonstrated absence of flow in a venous sinus/cortical vein and intraluminal venous thrombus^2,3,10,11^. The authors classified the configurations of CVST into four types for the ease of discussion (Fig. 2): type A (partial thrombosis of the sinus), type B (complete thrombosis of the sinus without cortical vein involvement), type C (cortical vein thrombosis only), and type D (complete thrombosis of the sinus with cortical vein involvement). The lengths of the thrombus in the sinuses were measured on MRV and digital subtraction angiography (DSA) by imaging software (SmartIris and UltraQueryEx, Taiwan Electronic Data Processing Co., Taiwan). CTV was not used for measurement because imaging artifacts at the posterior fossa might compromise the accuracy. All of the radiological studies were reviewed and agreed upon by the senior authors (YST and WHC).

### Endovascular procedures

The procedure was performed under general anesthesia. Seldinger technique was used to establish access to the femoral artery, and an 8 Fr vascular sheath was inserted into the femoral vein. Diagnostic cerebral DSA and DSV were performed to reveal the length and location of the thrombus. The devices for endovascular mechanical thrombectomy (EMT) and on-site chemical thrombolysis (OCT) included a large-bore suction catheter, a balloon catheter, and/or a stent retriever. First, a large-bore catheter was navigated to the proximal portion of the thrombi under the guidance of a microwire or a coaxial microcatheter system. The microcatheter system was gradually advanced to the distal portion of the thrombi to confirm the total length of the thrombosis. Direct aspiration technique was performed through the large-bore catheter extending as distal as possible. A balloon catheter was used to anchor the large-bore catheter and to disrupt the clots. At the same time, OCT with urokinase was performed (intrasinus slow bolus injection, 120,000 to 600,000 IU)^9,12^. After the infusion of urokinase, the patency of the sinus was checked with cerebral DSA/DSV. When acceptable recanalization was noticed, the large-bore catheter was left inside the sinus for continuous OCT with urokinase for 1–2 days (25,000 IU/h, usually 24 hours, depending on the degree of recanalization)^13^.

### Follow-Up protocols

Cone-beam CT was performed right after the endovascular procedure, and the plain CT was arranged the next day to rule out immediate new-onset/progression of ICH. Before discharge, DSA/DSV and MRA/MRV were repeated. The criteria for defining recanalization were as follows: (1) complete recanalization when all occluded sinuses/cortical veins were patent; (2) partial recanalization when one or more occluded sinuses/cortical veins showed improved flow or visualization of branches; and (3) no recanalization when all the occluded sinuses/cortical veins failed to achieve any degree of recanalization. During hospitalization, plain CT of the brain was performed when the patients’ clinical condition deteriorated. The safety outcomes of primary interest of the endovascular treatment for CVST patients included intra-procedural rupture/laceration of the sinus and new-onset/progression of symptomatic ICH after the procedure. Heparin (UFH or LMWH) was continued during hospitalization, and all the patients took warfarin after discharge. The international normalized ratio (INR) level was maintained within 2–3. Four-dimension MRA/MRV was arranged 3 months after discharge.

### Statistical analysis

Categorical variables were analyzed by using the chi-squared test; continuous variables by the Mann–Whitney U test. The median and interquartile ranges (IQR) were also presented. A 5% significance level (two-tailed) was accepted for hypothesis testing. The SPSS statistical software package (version 22.0; International Business Machines Corp, Armonk, NY, USA) was used.

### Ethical approval

All procedures performed in studies involving human participants were in accordance with the ethical standards of the Institutional Review Board I&II of Taichung Veterans General Hospital (IRB TCVGH No.: CE17084A) and with the 1964 Helsinki declaration and its later amendments or comparable ethical standards. For this retrospective study, formal consents were not required.

## Results

### Demographic data

From 2005 to 2015, a total thirty patients (14 males and 16 females) with CVST were included. Sixteen patients (8 males and 8 females) were in I/S group; fourteen patients (6 males and 8 females) belonged to D group. (Of note, among patients in D group, none of the patient/parent/guardian/next of kin refused the endovascular therapy as a salvage treatment.) All of the patients received heparin treatment after the confirmation of CVST diagnosis: two patients (one in each group) received UFH, and the rest received LMWH. Because our institute is a tertiary referral center, some of the CVST patients had received heparin treatment in outside hospitals in this study. From available records in D group, the mean time frame from the start of heparin treatment to the endovascular treatment was 3.2 days (38 days/12 patients), during which these patients continued to deteriorate clinically despite heparin treatment. The demographic data of I/S group and D group were in Table 1. There were no differences in gender, age, risk factors, initial ICH size, length of thrombosis, and CVST configurations between the two groups. All patients in D group had initial complete stenosis of the sinuses (type B or type D), with higher initial mRS (p = 0.006), lower initial GCS (p = 0.007), and more seizures (p = 0.031), which reflected the disease severity and presumable bad outcomes. Two or more sinuses were involved in 12 patients (40%). Transverse sinus (19/30) and superior sagittal sinus (SSS) (13/30) were the most affected sinuses. D group had more SSS involvement (p = 0.001). Headaches were the most common presenting symptom (76.7%). In this series, the most common CVST configuration was type D (46.7%), followed by type B (40%), type A (6.7%) and type C (6.7%).

**Table 1:** Demographic data and CVST characteristics in clinical deteriorating patients after heparin treatment (D group, with subsequent EMT/OCT) and clinical improving/stable patients (I/S group).

	D group (w/subsequent EVT/OCT) (n = 14)		I/S group (n = 16)		*p* value
	n	%	n	%	
**Gender**					0.980
female	8	(57.1%)	8	(50.0%)	
male	6	(42.9%)	8	(50.0%)	
**Age (yrs)**	47.50	(29.75–54.25)	40.00	(34.25–46.75)	0.677
**Risk factors**					0.186
coagulation dysfunction	6	(42.9%)	4	(25.0%)	
autoimmune diseases	2	(14.3%)	3	(18.8%)	
malignancy	2	(14.3%)	0	(0.0%)	
idiopathic	3	(21.4%)	6	(37.5%)	
pregnancy	1	(7.1%)	0	(0.0%)	
medications	0	(0.0%)	3	(18.8%)	
**Symptoms & Signs**					
headaches	9	(64.3%)	14	(87.5%)	0.204
seizures	6	(42.9%)	1	(6.3%)	0.031*
focal deficits	7	(50.0%)	7	(43.8%)	1.000
**Onset to Diagnosis (days)**	2.00	(1.00–3.25)	7.00	(2.00–13.00)	0.011*
**Initial mRS**	3.00	(1.00–4.00)	1.00	(1.00–2.00)	0.006**
**Initial GCS**	13.50	(10.25–14.25)	15.00	(14.00–15.00)	0.007**
**Initial ICH size**					0.674
nil	9	(64.3%)	10	(62.5%)	
<3 cm	2	(14.3%)	4	(25.0%)	
≧3 cm	3	(21.4%)	2	(12.5%)	
**Location of Thrombosis**^**&**^					
SSS	11	(78.6%)	2	(12.5%)	0.001**
TS	6	(42.9%)	13	(81.3%)	0.072
SiS	3	(21.4%)	4	(25.0%)	1.000
StS	0	(0.0%)	1	(6.3%)	1.000
cortical veins	9	(64.3%)	7	(43.8%)	0.448
**CVST Configurations**					0.147
type A	0	(0.0%)	2	(12.5%)	
type B	5	(35.7%)	7	(43.8%)	
type C	0	(0.0%)	2	(12.5%)	
type D	9	(64.3%)	5	(31.3%)	
**Length of thrombosis (cm)**	9.56	(7.13–12.35)	8.02	(4.71–10.46)	0.382
**Length (group)**					0.429
<10 cm	8	(57.1%)	9	(75.0%)	
**≥**10 cm	6	(42.9%)	3	(25.0%)	
**Discharge GCS**	15.00	(15.00–15.00)	15.00	(15.00–15.00)	0.212
**Discharge mRS**	0.00	(0.00–1.00)	0.00	(0.00–0.75)	0.504
**Length of Hospitalization (day)**	14.00	(8.75–17.00)	16.00	(9.25–19.75)	0.349
**3-months mRS**	0.00	(0.00–1.00)	0.00	(0.00–0.00)	0.501
**3-months mRS (group)**					1.000
mRS = 0–1	12	(85.7%)	14	(87.5%)	
mRS = 2–6	2	(14.3%)	2	(12.5%)	
**Recanalization**^**#**^ **(MR at 3 mo f/u)**					0.706
failed	1	(7.1%)	2	(15.4%)	
partial	7	(50.0%)	7	(53.8%)	
complete	6	(42.9%)	4	(30.8%)	

Chi-square test. Mann-Whitney U test, Median (IQR). *p < 0.05, **p < 0.01.

^&^Two or more sinuses were involved in 12 patients, so the sums of the percentages were more than 100%.

^#^Three patients in I/S group did not have MR study at 3 months follow-up.

abbreviations: clinical deteriorating patients = D group; clinical improving/stable patients = I/S group; endovascular mechanical thrombectomy = EMT; on-site chemical thrombolysis = OCT; sigmoid sinus = SiS; straight sinus = StS; superior sagittal sinus = SSS; transverse sinus = TS.

### Clinical outcomes & statistical analysis

**Figure 3:**
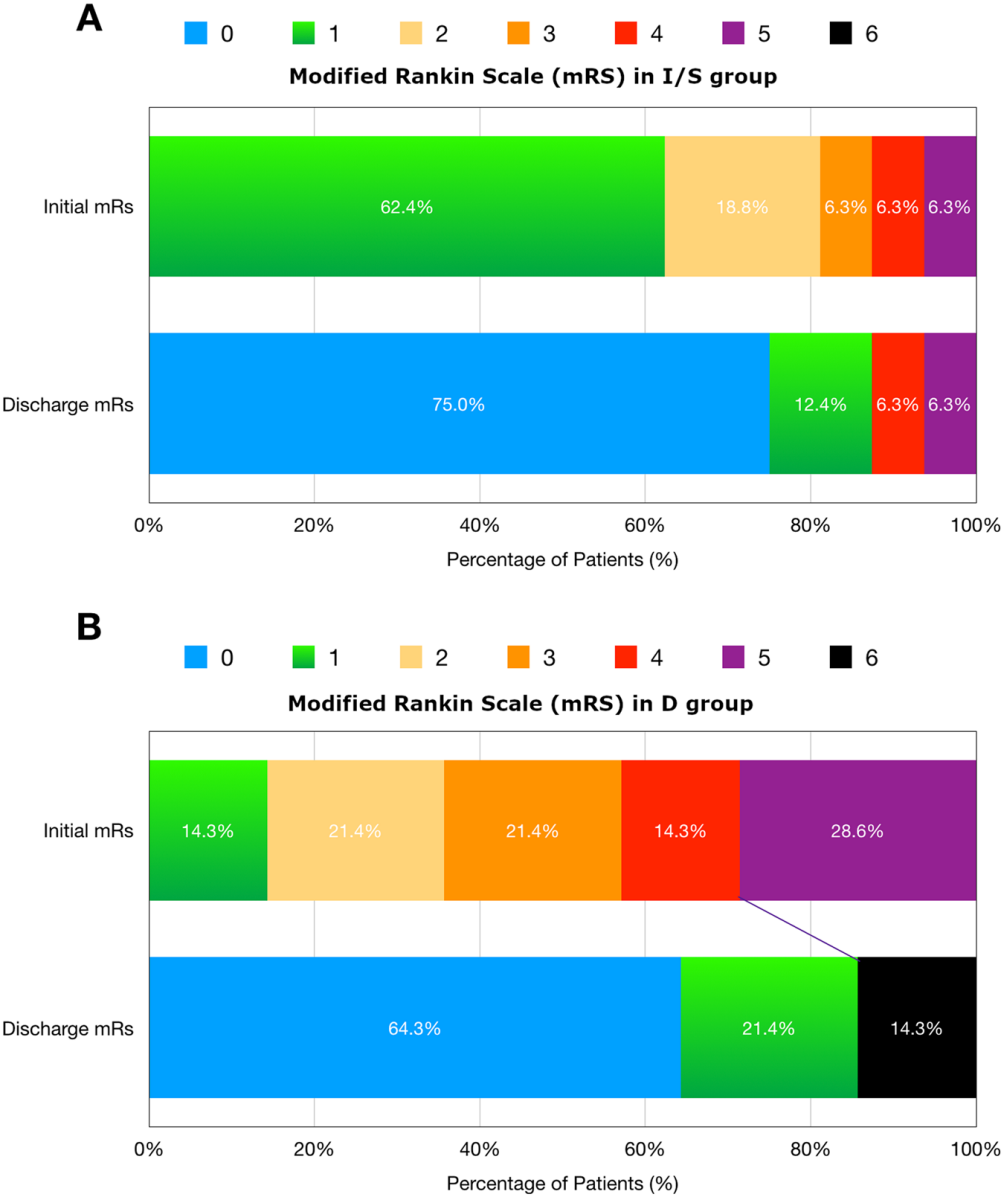
Percentages of CVST patients in each mRS at initial presentation and at discharge in (**A**) I/S group, clinical improving/stable and (**B**) D group, clinical deteriorating with subsequent endovascular treatment. Categorical shifts were demonstrated. In statistical analysis, compared with I/S group, the patients in D group had higher initial mRS (p = 0.006), but there were no significant differences in mRS at discharge between the two groups (p = 0.504).

Summary of clinical/radiographic findings and treatment details in I/S group and D group were in Supplementary Tables 1 and 2, respectively. In this study, 86.7% (26/30) of the patients had good outcomes at 3-months follow up (mRS = 0–1); 6.7% (2/30) had bad outcomes (mRS = 2–5); the overall mortality was 6.7% (2/30). The categorical shifts of the patients’ mRS at initial presentation and at discharge in I/S group and D group were demonstrated in Fig. 3. Compared with I/S group, D group had more severe clinical conditions at initial presentation, but there were no significant differences in mRS at discharge, GCS at discharge, and mRS at 3 months between the two groups (p = 0.504, 0.212, and 0.501, respectively). (Table 1) Shorter length of clot burden (<10 cm) and lower initial mRS (or higher initial GCS) were important factors associated with better outcomes [p = 0.008 and p = 0.003 (0.004), respectively]. Thrombosis involving SSS was associated with bad outcomes (p = 0.026). (Table 2)

**Table 2:** Statistical analysis of demographic/clinical data and CVST characteristics between patients with good (mRS = 0–1) and bad (mRS = 2–6) outcomes.

	mRS 0–1 (n = 26)		mRS 2–6 (n = 4)		*p* value
	n	%	n	%	
**Gender**					1.000
female	14	(53.8%)	2	(50.0%)	
male	12	(46.2%)	2	(50.0%)	
**Age (yrs)**	40.00	(33.50–48.50)	69.50	(33.50–86.00)	0.082
**Risk Factors**					0.659
coagulation dysfunction	9	(34.6%)	1	(25.0%)	
autoimmune diseases	4	(15.4%)	1	(25.0%)	
malignancy	1	(3.8%)	1	(25.0%)	
idiopathic	8	(30.8%)	1	(25.0%)	
pregnancy	1	(3.8%)	0	(0.0%)	
medications	3	(11.5%)	0	(0.0%)	
**Symptoms & Signs**					
headaches	21	(80.8%)	2	(50.0%)	0.225
seizures	5	(19.2%)	2	(50.0%)	0.225
focal deficits	11	(42.3%)	3	(75.0%)	0.315
**Onset to Diagnosis (day)**	3.00	(2.00–7.25)	3.50	(2.25–6.25)	0.877
**initial mRS**	2.00	(1.00–3.00)	5.00	(4.25–5.00)	0.003**
**initial GCS**	15.00	(14.00–15.00)	11.00	(5.00–11.75)	0.004**
**Length of Hospitalization (day)**	14.00	(8.75–18.25)	20.00	(10.75–28.50)	0.221
**Initial ICH size**					0.188
nil	18	(69.2%)	1	(25.0%)	
<3 cm	4	(15.4%)	2	(50.0%)	
≧3 cm	4	(15.4%)	1	(25.0%)	
**Location of Thrombosis**^**&**^					
SSS	9	(34.6%)	4	(100.0%)	0.026*
TS	18	(69.2%)	1	(25.0%)	0.126
SiS	7	(26.9%)	0	(0.0%)	0.548
StS	1	(3.8%)	0	(0.0%)	1.000
cortical veins	13	(50.0%)	3	(75.0%)	0.602
**CVST Configurations**					0.644
type A	2	(7.7%)	0	(0.0%)	
type B	11	(42.3%)	1	(25.0%)	
type C	2	(7.7%)	0	(0.0%)	
type D	11	(42.3%)	3	(75.0%)	
**Length of Thrombosis (cm)**	8.02	(4.73–9.90)	14.75	(11.13–20.07)	0.009**
**Length Group**					0.008**
<10	17	(77.3%)	0	(0.0%)	
**≥**10	5	(22.7%)	4	(100.0%)	
**Recanalization**^**#**^ **(MR at 3 mo f/u)**					0.235
failed	2	(8.3%)	1	(33.3%)	
partial	12	(50.0%)	2	(66.7%)	
complete	10	(41.7%)	0	(0.0%)	

Chi-square test. Mann-Whitney U test, Median (IQR). *p < 0.05, **p < 0.01.

^&^Two or more sinuses were involved in 12 patients, so the sums of the percentages were more than 100%.

^#^Three patients in I/S group did not have MR study at 3 months follow-up.

abbreviations: sigmoid sinus = SiS; straight sinus = StS; superior sagittal sinus = SSS; transverse sinus = TS.

**Figure 4:**
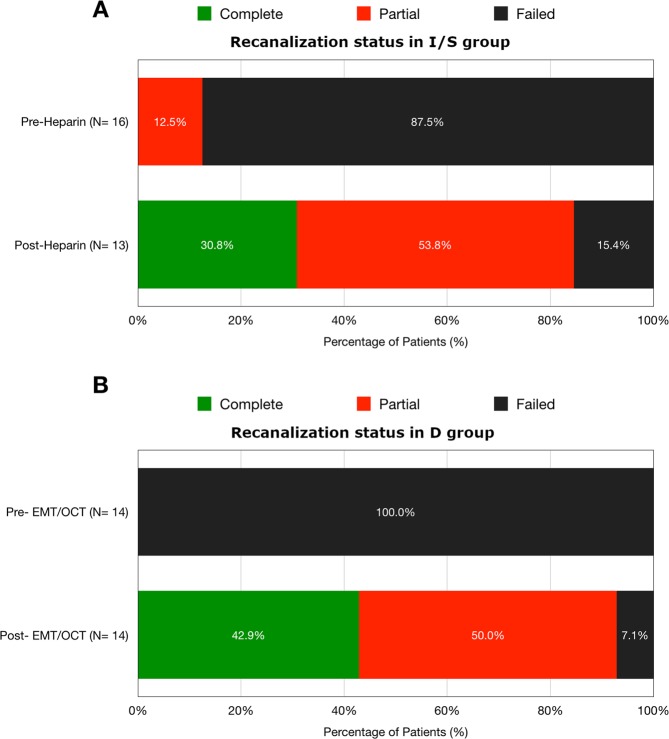
Percentages of the recanalization status in pre- and post-treatment in (**A**) I/S group, clinical improving/stable and (**B**) D group, clinical deteriorating with subsequent endovascular treatment. In I/S group, failed recanalization rate was higher than that of D group. In D group, all patients had total thrombosis of the sinuses at admission, and comparable recanalization rate was achieved at the end of the study.

In I/S group, thirteen patients had available MRV at 3-months follow up for analysis, and the recanalization status (failed/partial/complete) before and after the heparin treatment revealed: (1) failed/partial → complete in 30.8%, (2) failed → partial in 53.8%, and (3) failed → failed in 15.4%; in D group, all patients had MR study at 3 months follow-up, and the recanalization status revealed: (1) failed → complete in 42.9%, (2) failed → partial in 50.0%, and (3) failed → failed, 7.1%. (Fig. 4) In I/S group, failed recanalization rate was higher than that of D group. In D group, all patients had total thrombosis of the sinuses at admission, and comparable recanalization rate was achieved at the end of the study.

At initial presentation, 11 (36.7%) CVST patients had ICHs, and worsening symptomatic ICHs were found in 2 cases (6.7%), one in each group, managed surgically (decompressive craniectomy). Two patients died in D group: Patient #4 had no recanalization and died of ICH progression despite additional surgical treatment; Patient #10 died of sudden incurable pulmonary embolism. (Supplementary Table 2) The overall mortality of the study was 6.7% (2/30). There were no catheter-related complications (e.g., catheter-tip fracture, groin/retroperitoneal hematoma, and sinus perforation) noticed.

## Discussion

CVST is a rare cause of cerebral infarctions that accounts for approximately 0.5–1% of all strokes^14^. UFH or LMWH is currently the first-line treatment for patients with CVST during hospitalization^2,3,15–17^. Anticoagulation therapy can prevent the extension of thrombosis and dissolve the thrombus in the sinuses and cortical veins. However, the dosage needed to be effective at the nidus might not be enough through systemic use. According to the ISCVT study, about 13% of patients still had bad outcomes (mRS = 2–5), and the mortality (mRS = 6) rate was 8.3%; once the patient survived the acute phase, long-term prognosis was generally satisfactory^4^. However, questions about the safety and validity of the endovascular treatment plus heparin for CVST over the heparin treatment alone in the acute phase have not been answered^9,12,18–20^. In the literature, a subgroup of CVST patients who harbored risk factors known for poor prognosis and mortality (e.g., decreased level of consciousness, coma, and seizure activity) were oftentimes unresponsive to systemic heparin treatment in the acute phase^4,5,7–9^. In this study, all of the patients in D group had initial complete stenosis of the sinuses (type B or type D), with higher mRS (p = 0.006), lower GCS (p = 0.007), and more seizures (p = 0.031) at initial presentation, which reflected the disease severity and high risks of poor outcome and mortality in the acute phase^4,5,7–9^. In I/S group, patients were responsive to heparin with milder initial presentation. However, there were no significant differences in the mRS/GCS at discharge and the mRS at 3 months between D group and I/S group (p = 0.504/0.212 and p = 0.501, respectively). In this study, 6.7% of the patients had bad outcomes (mRS = 2–5), and the overall mortality (mRS = 6) was 6.7%. The authors demonstrated the safety of the endovascular treatment and its possible validity through an indirect method in this study because of the very low incidence of CVST. Although the endovascular treatment (thrombolysis and/or thrombectomy) has not been recommended in the treatment guidelines for CVST^2,3^., in the authors’ opinion, severe CVST patients who are unresponsive to heparin might benefit from the endovascular treatment as a salvage treatment in the acute phase.

Shorter length of thrombosis was an important factor associated with better outcomes (p = 0.009). The main reason that CVST patients continue to deteriorate neurologically is cerebral venous congestion without adequate venous drainage compensation, which is secondary to undissolved thrombi. Clot burden is the only factor that we can change the disease course by endovascular treatment. Our results demonstrated that EMT and OCT might be a suitable alternative for heparin non-responders of CVST. EMT removed and disrupted the thrombi directly, thus increasing the surface area of the thrombi for OCT activity. The catheter was left in the sinus for continuous OCT for 24–48 hours without increased rates of new-onset/progression of ICHs. In current study, the status of recanalization (failed, partial, or complete) could not be statistically related to clinical outcomes because the case number was not big enough for further subgroup analysis or for adjustments of the confounding factors. In the literature, complete recanalization may influence the functional outcome, although the results were still inconclusive^17,21^. One study showed that failed recanalization of CVST after the endovascular treatment was 4.7%, a condition with a high mortality rate (83%)^20^. CVST patients who failed to have recanalization could survive the acute phase possibly due to small clot burden and good collateral outflows, especially in type C patients. Symptoms of CVST such as headaches and vomiting could improve after partial recanalization^22,23^, which was also observed in our patients.

The authors proposed a classification to describe four types of CVST configurations: type A (partial thrombosis of the sinus), type B (complete thrombosis of the sinus without cortical vein involvement), type C (cortical vein thrombosis only), and type D (complete thrombosis of the sinus with cortical vein involvement). (Fig. 2) Type D CVST patients were the most common (46.7%) in our series and tended to deteriorate clinically. Nine out of thirteen type D CVST patients ended up in D group. In the literature, this type of patients was related to worse outcomes^24^. Type B CVST patients were the second most common (40%). In type D and type B CVST, EMT and OCT could achieve rapid recanalization (either complete or partial) in the sinus. The authors did not use balloon angioplasty intentionally to expand the thrombosed SSS, in order to avoid pushing clots into the cortical veins^25^. In type A CVST, the symptoms tended to be milder. Hence, systemic anticoagulation would suffice, and there were no type A patients in D group. In Type C CVST, which is less common, the presentation is different from that of other types. There are less increased intracranial pressures and headaches but more venous infarctions, localized edema, and ICH^24^. There were no type C patients in D group, either. EMT is not a safe treatment option for type C CVST because cortical veins are fragile and easy to be perforated under manipulation.

Stent retrievers were not usually used for clot disruption in CVST patients in our institute. Although newer stent retrievers for acute ischemic stroke (AIS) have been developed for years, the use of newer devices for CVST did not significantly affect outcomes nor complete recanalization^20^. The diameter of a stent retriever for AIS is usually smaller than the horizontal diameter of the SSS at the level of the coronal suture (about 6 mm), and usually the more posterior the larger the diameter of the SSS^26^, which shows an obvious diameter discrepancy. The venous sinus channels to which cortical bridging veins connect also vary in sizes. During EMT with a stent retriever, the walls of the venous sinuses or bridging veins at the connection sites might get damaged or torn. In the authors’ opinion, a stent retriever is not the first choice for CVST when other inexpensive alternatives are handy.

### Study limitations

This is a retrospective study. Due to the rarity of CVST, the endovascular treatment is reserved for CVST patients who are heparin non-responders in our institute. Hence, there is no randomization in this study. Some patients were referred from local or regional hospitals, so the time from onset to diagnosis of CVST might be affected. Although the length of thrombosis and the involvement of SSS were associated with poor outcomes, small patient size limited our ability to perform adjustment of confounding factors or further subgroup analysis (e.g. status of recanalization, CVST configurations, etc.) The proposed classification system for CVST configurations could be used in further studies to help clarify the effectiveness of treatment among different types. Despite the possible advantages of EMT and OCT, there is still no consensus on the type of thrombolytic drugs, dose, or admission rate (bolus injection/continuous infusion with a catheter) because of the lack of adequate numbers of studies in this area. It is a pity that the TO-ACT study^12^, an international, multicenter prospective, randomized, open-label, blinded endpoint trial, was terminated in advance. In this trial, there was no mandatory/standard protocol for the endovascular procedures and the usage of thrombolytic agents, which we considered a difficulty for statistical analysis. Multi-center collaborative works would still be needed for prospective, comparative, randomized trials to rigorously assess the outcomes of the endovascular treatment for CVST.

## Conclusion

This study identified that the clot burden (>10 cm length) and its location (involving the SSS) were associated with poor outcomes. Combined endovascular mechanical thrombectomy and on-site chemical thrombolysis after heparin treatment for severe CVST were reasonably safe. Endovascular treatment might be considered as a salvage treatment in severe CVST patients who are unresponsive to heparin with heavy clot burden involving SSS in the acute phase. However, further studies are needed to confirm its efficacy and validity.

## Supplementary information

Supplementary Tables 1 and 2.

## Data Availability

Raw data were generated in Taichung Veterans General Hospital. Derived data supporting the findings of this study are available from the corresponding author Yang, Tsuei, and Shen on request.
