# Supplementary material for: Endovascular Mechanical Thrombectomy and On-Site Chemical Thrombolysis for Severe Cerebral Venous Sinus Thrombosis
Source: Sci Rep. 2020 Mar 18;10:4937. doi: 10.1038/s41598-020-61884-5 (PMC7080812; doi:10.1038/s41598-020-61884-5)
Supplement: Supplementary file 1 — Supplementary Tables 1 and 2. [file 41598_2020_61884_MOESM1_ESM.pdf]

Endovascular Mechanical Thrombectomy and On-Site Chemical Thrombosysis for Severe Cerebral Venous Sinus Thrombosis

Chih-Hsiang Liao[1,2,3#], M.D., Nien-Chen Liao[4#], M.D., Wen-Hsien Chen[5#], M.D., Hung-Chieh Chen,[5,6] M.D., Chiung-Chyi Shen,[2,7*] M.D., Shun-Fa Yang[1,8*], Ph.D., Yuang-Seng Tsuei[1,2,7*], M.D.

[1]Institute of Medicine, Chung Shan Medical University, Taiwan
[2]Department of Neurosurgery, Neurological Institute, Taichung Veterans General Hospital, Taiwan
[3]Department of Critical Care Medicine, Taichung Veterans General Hospital, Taiwan
[4]Department of Neurology, Neurological Institute, Taichung Veterans General Hospital, Taiwan
[5]Division of Neuroradiology, Department of Radiology, Taichung Veterans General Hospital, Taiwan
[6]School of Medicine, National Yang-Ming University, Taiwan
[7]Department of Neurosurgery, Tri-service General Hospital, National Defense Medical Center, Taiwan
[8]Department of Medical Research, Chung Shan Medical University Hospital, Taichung, Taiwan

# These authors have contributed equally to this article.

Supplementary Tables 1 & 2

Supplementary Table 1. Summary of clinical/radiographic findings and treatment details in clinical improving/stable patients after heparin treatment (I/S group).

Patient No.	Sex	Age	Risk factors	Onset to diagnosis (day)	initial / discharge GCS	initial / 3-months mRS	CVST config. (type)	location / length (cm) of thrombosis*	ICH at presentation[$]	recanalization (MR: initial → 3 mo f/u)
**1**	F	23	antiphospholipid syndrome protein C/S deficiency	14	15 / 15	2 / 0	A	Rt TS / N/A	-	partial → complete
**2**	F	26	protein C/S deficiency	20	15 / 15	1 / 0	B	Lt TS / 4.20	-	no MR
**3**	M	52	Churg-Strauss syndrome	60	15 / 15	1 / 1	B	Lt TS & SiS / 7.73	-	failed → partial
**4**	M	35	polycythemia	17	14 / 15	1 / 0	A	Lt TS & JV / N/A	-	partial → complete
**5**	M	42	idiopathic	2	14 / 15	1 / 0	D	Lt TS & SiS / 9.21	++, Lt temporal	failed → partial
**6**	F	41	idiopathic	7	15 / 15	1 / 0	B	Rt TS / 4.71	-	no MR
**7**	M	36	idiopathic	4	15 / 15	1 / 0	C	parasagittal parietal cortical veins / N/A	+, Lt frontal	failed → complete
**8**	F	39	contraceptive pills	10	15 / 15	1 / 0	D	StS & Lt TS / 12.87	-	failed → partial
**9**	F	47	protein C/S deficiency	2	15 / 15	2 / 0	B	Rt TS & SiS / 7.35	-	failed → partial
**10**	F	35	contraceptive pills	7	15 / 15	1 / 0	B	Lt TS & SiS / 8.98	-	failed → partial
**11**	F	45	protein C/S deficiency	2	14 / 15	1 / 0	D	Lt TS & SiS / 8.31	++, Lt temporal	failed → failed
**12**	M	90	idiopathic	4	11 / 12	4 / 5	B	lower 1/3 SSS & Lt TS / 10.87	+, Lt parietal	no MR
**13**	F	46	idiopathic	8	15 / 15	2 / 0	D	Lt TS & cortical veins / 4.74	+, Lt temporal	failed → failed
**14**	M	31	amphetamine abuser venous angioma	1	15 / 15	3 / 0	C	cortical veins / N/A	-	failed → complete
**15**	M	65	ANA positive HLA B27 positive	7	12 / 13	5 / 4	D	SSS & Lt cortical veins / 17.58	+ → ++ & OP, Lt temporal	failed → partial
**16**	M	34	idiopathic	2	15 / 15	1 / 0	B	Lt TS / 4.55	-	failed → partial

* The length of thrombosis was measured by calculating the total lengths of complete sinus thrombosis. Hence, partial thrombosis and/or cortical vein thrombosis (type A and type C) were excluded.

[&] The size of ICH: none = -; < 3 cm = +; > 3 cm = ++.

abbreviations: endovascular mechanical thrombectomy = EMT; jugular vein = JV; on-site chemical thrombolysis = OCT; sigmoid sinus = SiS; straight sinus = StS; superior sagittal sinus = SSS; transverse sinus = TS.

Supplementary Table 2. Summary of clinical/radiographic findings and treatment details in clinical deteriorating patients after heparin treatment (D group).

Patient No.	Sex	Age	Risk factors	Onset to diagnosis (day)	initial / discharge GCS	initial / 3-months mRS	CVST config. (type)	location / length (cm) of thrombosis*	ICH at presentation[&]	Treatment[#]	recanalization MR: initial → 3 mo f/u
1	M	55	antiphospholipid syndrome protein C/S deficiency	2	13 / 15	3 / 0	D	Rt TS & SSS / 9.45	-	EMT / OCT (UK: 360K U)	failed → partial
2	F	36	pregnancy	7	14 / 15	3 / 0	B	SSS / 7.30	+, Rt parietal	EMT / OCT (UK: 360K U)	failed → complete
3	M	34	idiopathic	4	15 / 15	2 / 0	B	SSS / 17.18	-	EMT / OCT (UK: 720K U)	failed → complete
4[$]	F	74	gastric cancer	2	3 / died	5 / 6	D	SSS & bilateral TS / 20.9	++ → worsening & OP , Rt fronto-temporo-occipital & Lt occipital	EMT / OCT (UK: 600K U)	failed → failed
5	F	54	idiopathic	2	12 / 15	4 / 0	B	Lt TS / 3.3	+, basal cistern SAH	EMT / OCT (UK: 180K U)	failed → partial
6	M	32	idiopathic thrombocytosis	3	15 / 15	2 / 0	B	SSS, Lt TS & StS / 10.62	-	EMT / OCT (UK: 300K U)	failed → partial
7	M	21	idiopathic	3	12 / 15	4 / 0	B	Lt TS & SiS / 9.66	++, Lt temporal	EMT / OCT (UK: 540K U)	failed → complete
8	F	48	antiphospholipid syndrome	1	14 / 15	2 / 0	D	mid SSS / 8.74	-	EMT (+Penumbra) / OCT (UK: 720K U)	failed → partial
9	F	51	protein C/S deficiency	1	8 / 15	5 / 1	D	lower 1/2 SSS & Rt TS / 11.74	-	EMT / OCT (UK: 480K U) +catheter retention (UK: 600K U/ day for 2 days)	failed → complete
10	F	23	protein C/S deficiency	3	11 / died	5 / 6	D	SSS / 11.92	-	EMT / OCT (UK: 480K U) +catheter retention (UK: 600K U/ day for 2 days)	failed → partial
11	F	47	protein C/S deficiency	2	14 / 15	1 / 0	D	Lt TS & vein of Labbé / 7.70	-	EMT + OCT (UK: 120K U)	failed → complete
12	F	63	lung cancer	1	14 / 15	3 / 1	D	SSS & SiS / 3.83	-	EMT / OCT (UK: 240K U) +catheter retention (UK: 600K U/ day for 1 days)	failed → partial
13[$]	M	17	protein C/S deficiency	1	3 / 15	5 / 0	D	SSS / 13.64	++, Lt fronto-parietal	EMT (+stent retriver) / OCT (UK: 120K U)	failed → partial

**14**	M	50	Sjogren's syndrome protein C/S deficiency	7	15 / 15	1 / 0	D	lower 1/3 SSS / 6.63	-	EMT (+Penumbra) / OCT (UK: 480K U)	failed → complete

* The length of thrombosis was measured by calculating the total lengths of complete sinus thrombosis. Hence, partial thrombosis and/or cortical vein thrombosis (type A and type C) were excluded.

& The size of ICH: none = -; < 3 cm = +; > 3 cm = ++.

\# EMT was performed with a large-bore catheter for direct aspiration technique and a balloon to disrupt the clots. OCT was carried out with urokinase injected through the microcatheter distally and the large-bore catheter proximally in the sinus. Whether the large-bore catheter was left in place for subsequent continuous urokinase injection was determined according to the effectiveness of OCT by the treating interventionist.

$ Patient #4 and #13 had generalized tonic clonic seizures at initial presentation. Other patients who had full scores of GCS at initial presentation received the endovascular treatment due to epilepsia partialis continua despite anti-epileptic medications.

abbreviations: endovascular mechanical thrombectomy = EMT; jugular vein = JV; on-site chemical thrombolysis = OCT; sigmoid sinus = SiS; straight sinus = StS; superior sagittal sinus = SSS; transverse sinus = TS; urokinase = UK.
